# Evaluation of Caries-Free Restorations Bonded with Various Adhesive Systems: *In Vitro* Study

**DOI:** 10.1155/2020/5859835

**Published:** 2020-07-22

**Authors:** Morakot Piemjai, Pareewan Chantarawej, Nobuo Nakabayashi

**Affiliations:** Department of Prosthodontics, Faculty of Dentistry, Chulalongkorn University, Bangkok, Thailand

## Abstract

**Purpose:**

Secondary caries originate from a leakage pathway where oral acids can penetrate faster and demineralize the tooth substrate deeper which can be visualized by dye penetration. The ability to prevent secondary caries by contemporary adhesive systems was evaluated in this study. Dye penetration distance through leakage and into the tooth substrate adjacent to Class V restorations after artificial caries exposure was compared.

**Materials and Methods:**

Previously frozen extracted human molars were used to prepare the Class V cavities at the CEJ on axial surfaces. All cavities were restored with either the resin-composite or amalgam with or without resin adhesives: dry bonding: Super-Bond D-Liner II Plus; moist bonding: All-Bond 2; and self-etch bonding: AQ Bond and Clearfil Protect Bond. Two subgroups of Super-Bond D-Liner II Plus were immersed for 14 days at 37°C either in artificial saliva (negative control) or the artificial caries solution. The other groups were soaked in the artificial caries solution. The distance of dye penetration into the adjacent enamel, cementum/dentin, and tooth-resin interfaces was measured after immersion in 0.5% basic fuchsin dye for 24 h. The tooth-resin interfacial layer was investigated using SEM.

**Results:**

No dye penetration into the tooth-resin interface was found in Super-Bond D-Liner II Plus and AQ Bond groups which demonstrated a constant hybrid layer after a chemical challenge. The leakage distance at the cementum/dentin-resin interface of All-Bond 2, Clearfil Protect Bond, and non-adhesive amalgam (positive control) groups was significantly higher than the distance of dye penetration into the adjacent demineralized root surface (*p* < 0.05).

**Conclusion:**

Caries associated with either amalgam or resin-composite restorations can be prevented using resin adhesives which can penetrate into the intact tooth substrate to form a stable hybrid layer. With caries-free restorations, tooth vitality may be conserved lifelong.

## 1. Introduction

Amalgam restorations have been used worldwide for more than 120 years because they are long lasting, low cost, and/or easy to manipulate. However, with the dark silver like color and the low levels of mercury vapor, they have been increasingly replaced with the tooth-colored resin-composite restorations. Hybridization of resin into the enamel or dentin creates a hybrid layer which provides the adhesion strength to the above cured resin adhesives [1]; therefore, with amalgam or resin-composite restorations coupled with this layer, it is not necessary to remove the sound tooth structure for mechanical retention or strength for long-term survival [2, 3]. This makes resin adhesives, which can provide a complete hybrid layer, one of the major contributors in conserving the tooth structure for life-long use.

In the last 40 years, dental adhesives have been developed from dry, wet or moist, to self-etch tooth conditioning to reduce clinical complications such as tooth hypersensitivity, secondary caries, pulpal infection, and/or restoration detachment. Many dentin-bonding adhesives such as a total-etch with either dry or moist techniques and self-etch systems have been introduced into the worldwide market to promote better retention and bond strength between the tooth-colored filling materials and the dentin substrate. Impermeable hybridized dentin prepared by penetrating a polymer network using 4-methacryloyloxyethyl trimellitate anhydride in methyl methacrylate initiated by the tri-*n*-butyl borane (4-META/MMA-TBB) resin into non-collagen collapsed dry bonding of demineralized dentin provides high tensile strength with cohesive failure occurring in resin and defect-free bonded specimens [4, 5]. This bonding technique has been clinically utilized since the 1980s. Tensile testing using mini-dumbbell specimens and a chemical challenge using HCl and NaOCl solutions have been shown to be effective in detecting defects such as remaining demineralized dentin, smears, and hybridized smear layer in the dentin-bonded specimens [5–7]. Very high microtensile bond strength in some wet or moist bonding techniques has been reported with mixed adhesive and cohesive failure; however, the quality of the hybrid layer was not well characterized except for nanoleakage and short-term degradation of the resin-dentin interfacial layer [8, 9]. Phosphoric acid conditioning of the dentin and moist bonding must be carefully controlled so as to not introduce permeable [10] and hydrolysable [11] demineralized dentin into the restored dentin which may then induce a wall lesion [12]. Simplification of the bonding steps from three- or two-step etch and rinse to two- or one-step self-etch by combining the etchant, primer, and/or bonding ingredients is highly effective in eliminating technical sensitivity while allowing easy manipulation just like for amalgam fillings. However, countering adverse effects of smears and hybridized smears using a self-etching system on reliable bonding for a complete seal is needed to prevent a leakage pathway [13–15].

One of the commonest failures in restored teeth is due to secondary caries. Fluoride ion-releasing adhesive material was reported for better caries prevention [16]. Nevertheless, secondary caries is still the major cause of failure of fluoride-releasing glass ionomer fillings [17, 18]. Also, in clinical studies, the incidence of secondary caries was not significantly reduced by the fluoride release from restorative materials [19]. Secondary caries may have been developed from leakage between the restorations and the tooth [20]. Visualization of the basic fuchsin dye, which easily binds to acidic GAGs in the demineralized dentin and accurately marks artificial root caries and initial wall lesions in the leakage pathway, suggests that a complete leakage-free hybrid layer can block lactic acid which is believed to be involved in the initial cause of caries [12]. Therefore, the ability to prevent secondary caries and complete microsealing, using a variety of commercial bonding adhesives, is simply demonstrated using artificial caries simulation and dye detection. The study hypothesis was that secondary caries could be inhibited by adhesive systems that could provide a stable hybrid layer with no leakage.

The objective of this study was to compare the effectiveness in preventing secondary caries of commercial adhesive systems. The dye penetration distance around the Class V resin-composite or amalgam restorations either using dry (Super-Bond D-Liner II Plus), moist (All-Bond 2), or self-etch (AQ Bond and Clearfil Protect Bond) bonding adhesives after artificial caries exposure was measured. The characteristics of the tooth-resin interface for each adhesive was analyzed using SEM.

## 2. Materials and Methods

The vital human molars that required extraction with the patients' signed informed consent were collected and frozen for less than 6 months. Teeth without cracks or caries were selected to prepare Class V cavities on axial surfaces at the CEJ using diamond burs (204, Intensiv, Grancia, Switzerland) with a high-speed hand piece under water spray. The box-form cavities approximately 2 mm high, 3 mm wide, and 1.5 mm deep with the occlusal margin in the enamel and the cervical margin in the cementum were randomly divided into 6 groups. Amalgam (Dispersalloy, DENTSPLY International Inc., DE, USA) restorations without adhesives (Non) were prepared for a positive control group. Four different bonding agents: dry (Super-Bond D-Liner II Plus (Sup; Sun Medical, Shiga, Japan)), moist (All-Bond 2 (All; Bisco Inc., IL, USA)), and self-etch (AQ-Bond (AQ; Sun Medical, Shiga, Japan) and Clearfil Protect Bond (Cle; Kuraray Medical Inc., Okayama, Japan)) were used for the remaining groups. The manipulation of adhesives followed the recommendations of the manufacturers (Table 1). Amalgam was used to restore in the Super-Bond D-Liner II Plus group, while the resin-composite (Metafil, Sun Medical, Shiga, Japan) was used in the other groups. After light-cured for 60 s or self-curing for 10 min, the resin-composite-restored margins were finished with fine diamond burs (4205, Intensiv, Grancia, Switzerland) with a high-speed hand piece, while fine white stone burs with a slow-speed hand piece were used to finish the amalgam-restored margins. Two subgroups of Super-Bond D-Liner II Plus were immersed for 14 days at 37°C either in artificial saliva (negative control) or 0.1 mol lactic acid in buffer solution (the artificial caries solution with a pH of 4.5) [12], while the other three groups: All-Bond 2, AQ-Bond, and Clearfil Protect Bond were soaked only in the artificial caries solution.

After cleaning and drying, all tooth surfaces were coated with two layers of nail varnish (Pias, Bangkok, Thailand), leaving the restoration and 1 mm above and below the enamel and cementum/dentin margins, respectively. All the samples were immersed in 0.5% basic fuchsin dye for 24 h prior to vertically sectioning through the middle of the cavities using a diamond disk and a micromotor hand piece to make two sections. The surface of each section was polished to be horizontally parallel using abrasive papers with the grit size #400 to #2,000. The distance of dye penetration into the adjacent enamel and cementum/dentin and the tooth-cured adhesive interface was measured using Image-Pro Plus software on a standardized image taken from a stereomicroscope attached to a digital camera (Nikon, Japan) at ×50, ×100, and ×200. Sectioned specimens with a crack line that influenced the dye penetration were excluded. The distance data were statistically analyzed using one-way ANOVA and multiple comparisons tests of the SPSS program at *p* < 0.05.

Four cavities of each bonding agents were prepared according to the method mentioned earlier to evaluate the tooth-resin interfacial layer. The sectioned specimens without epoxy embedding were sequentially abraded using the #400, #600, #800, #1,000, and #2,000 grit size silicon carbide paper and finished with 0.05 *µ*m alumina under wet conditions. One piece of each specimen was soaked into 6 mol/L HCl for 30 s, followed by 1% NaOCl for 60 min. The quality of the hybrid layer was investigated under SEM.

## 3. Results

**Figure 1:**
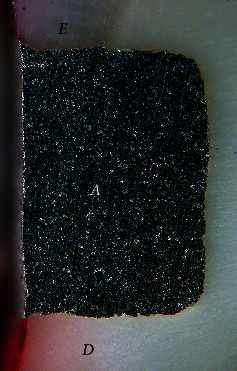
Leakage-free margin of amalgam bonded with Super-Bond D-Liner II Plus soaked in the artificial saliva, a negative control (original × 50, *E* = enamel, *D* = dentin, and *A* = amalgam).

**Figure 2:**
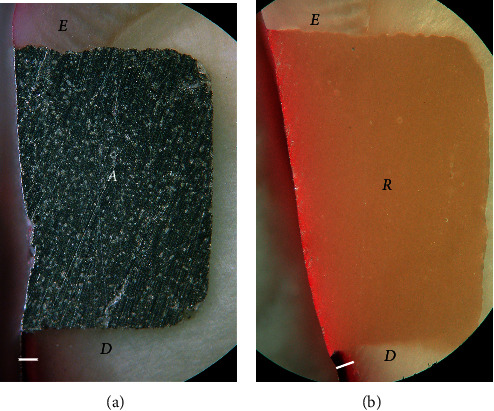
No dye penetration into the enamel- and cementum/dentin-resin interface and the adjacent enamel after artificial caries exposure: (a) amalgam bonded with Super-Bond D-Liner II Plus and (b) resin composite bonded with AQ-Bond (original × 50, *E* = enamel, *A* = amalgam, *D* = dentin, *R* = resin composite, and a white line = dye penetration into the adjacent cementum/dentin).

**Figure 3:**
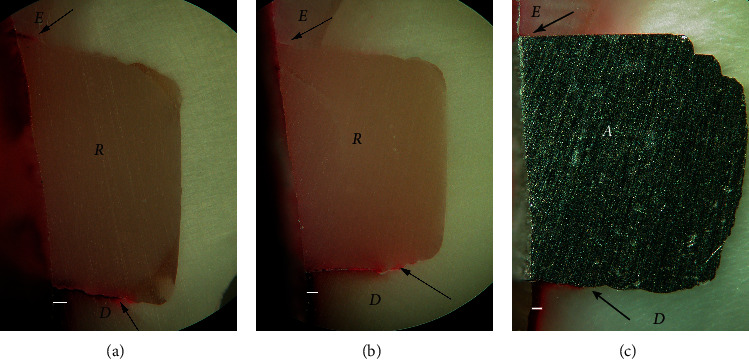
Dye penetration into leakage at the tooth-resin interface (arrowed) after artificial caries exposure: (a) All-Bond 2, (b) Clearfil Protect Bond, and (c) non-adhesive amalgam, a positive control (original × 50, *E* = enamel, *A* = amalgam, *D* = dentin, *R* = resin-composite, and a white line = dye penetration into the adjacent cementum/dentin).

Dye penetration distances (mean ± SD) through leakage and into the tooth substrate adjacent to Class V restorations for all groups are shown in Table 2. A leakage-free tooth-resin interface at the enamel and cementum/dentin margins was found in restorations coupled with Super-Bond D-Liner II Plus for both subgroups that were soaked in artificial saliva (negative control) and a lactic acid buffer and AQ-Bond specimens (Figures 1 and 2). Specimens in All-Bond 2, Clearfil Protect Bond, and non-adhesive amalgam (positive control) showed leakage both at the enamel- and cementum/dentin-resin interfaces (Figure 3).

No dye penetration into the adjacent enamel and cementum/dentin surfaces was found in all specimens soaked in artificial saliva (Sup, negative control), while specimens in all groups after artificial caries exposure showed dye penetration into the adjacent root surfaces with an average distance of 0.175 ± 0.039 mm (*n* = 48). One-way ANOVA found no significant difference in the distance of dye penetration into the adjacent cementum/dentin and leakage at the enamel- and cementum/dentin-resin interface among groups at *p* < 0.05. Pair-*t* test (*p* < 0.05) revealed significantly higher distances of leakage into the cementum/dentin-resin interface than that of the enamel-resin interface and dye penetration into the adjacent cementum/dentin in all leakage groups. No dye penetrated into the adjacent enamel after caries exposure for 14 days.

**Figure 4:**
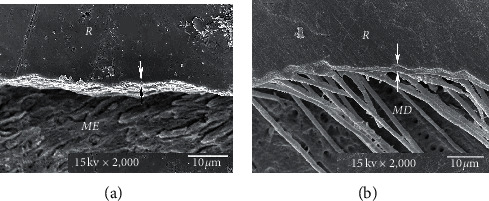
SEM micrograph of Super-Bond D-liner II Plus restored amalgam after chemical challenge, demonstrating the consistent and continuous thickness of the hybrid layer (≈3-4 *μ*m, arrowed) in the enamel (a) and dentin (b) (original × 2,000, *ME* = modified enamel, *MD* = modified dentin, and *R* = resin).

**Figure 5:**
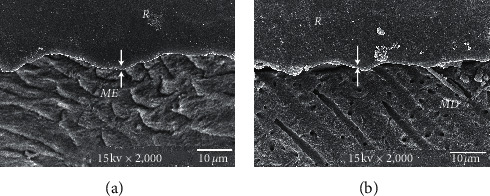
SEM micrograph of the AQ-Bond-restored resin composite after chemical challenge, demonstrating the consistent and continuous thickness of the hybrid layer (≈1-2 *μ*m, arrowed) in the enamel (a) and dentin (b) (original × 2,000, *ME* = modified enamel, *MD* = modified dentin, and *R* = resin).

**Figure 6:**
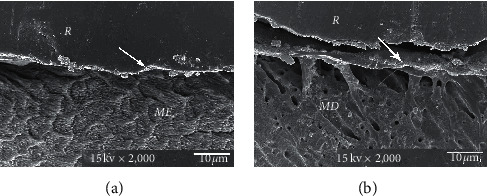
SEM micrograph of All-Bond 2-restored resin-composite after chemical challenge, demonstrating the detached and degraded interfacial layer (arrowed) in the enamel (a) and dentin (b) (original × 2,000, *ME* = modified enamel, *MD* = modified dentin, and *R* = resin).

**Figure 7:**
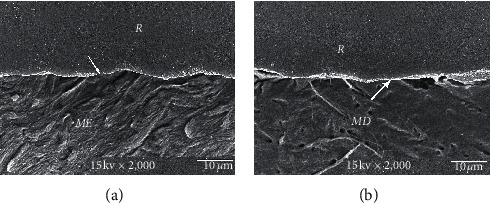
SEM micrograph of Clearfil Protect Bond-restored resin composite after chemical challenge, demonstrating the thin and degraded interfacial layer (arrowed) in the enamel (a) and dentin (b) (original × 2,000, *ME* = modified enamel, *MD* = modified dentin, and *R* = resin).

The consistent thickness of 3-4 *µ*m hybridized layer in Super-Bond D-Liner II Plus and a thin layer of approximately 1 *µ*m in AQ-Bond groups was demonstrated in both polished and chemically challenged specimens (Figures 4 and 5). A detached and degraded interfacial layer in All-Bond 2 specimens after soaking in HCl and NaOCl was observed (Figure 6). The interfacial layer after chemical immersion of the Clearfil specimens was not consistent and degraded (Figure 7).

## 4. Discussion

For many decades, microleakage as a critical component for initiation of a caries lesion under restorations has been reported [21, 22]. When a complete hybrid layer with a leakage-free interface was formed, there was no remaining demineralized tooth substrate or smear layer for dye or lactic acid to penetrate through [12, 14, 23]. The study results showed that amalgam restorations coupled with Super-Bond D-Liner II Plus provided the leakage-free enamel- and dentin-resin interfaces when soaked in artificial saliva and artificial caries solution (Figures 1 and 2(a)). This suggests that Super-Bond D-Liner II Plus using a 10 s etching period of 10% citric acid and 3% ferric chloride (10–3) conditioner, rinsed off and then air-dried for 10 s could prepare a tissue substrate permeable for HEMA and 4-META/MMA-TBB monomers which entirely impregnated and then polymerized to form an impermeable hybrid layer which resisted lactic acid penetration, the cause of secondary caries. A consistent thickness of 3-4 *µ*m hybrid layer after chemical challenge suggests that the high resin content could entirely protect and seal the tissue substrate (Figure 4). Encapsulated hydroxyapatite not demineralized by HCl in the hybridized dentin confirms that complete hybridization can be achieved in vital human teeth [24]. This assumes that amalgam-bonded and tooth-sealed restorations using this dry bonding system can prevent secondary caries formation in the oral cavity. This complete hybrid layer combined with resin-composite restoration also shows the ability to prevent initial wall lesions [12].

Resin-composite bonded with AQ-Bond could inhibit the marginal leakage at the tooth-resin interface after soaking in the lactic acid buffer solution (Figure 2(b)). AQ-Bond, the self-etch bonding agent, provided leakage-free tooth-resin interfaces by using monomer-soaked sponge scrubbing on the tooth surface for removal of a swollen smear layer, thus minimizing the adverse effect of a weak hybridized smear layer under the restoration [13, 14]. With two times scrubbing to remove all the smear layers, AQ-bond could effectively penetrate into the intact tooth substrate to form a high resin content 1-2 *µ*m hybrid layer (Figure 5), which could resist the penetration of the lactic acid buffer solution as well as the chemical challenge shown in this study.

The leakage of All-Bond 2, Clearfil, and non-adhesive amalgam specimens (Figure 3(a)–3(c), respectively) suggested a remaining of defect into which dye and lactic acid can penetrate [10, 14, 25]. All-Bond 2 using a phosphoric acid conditioner to remove the smear layer as same as to demineralize the underneath tooth substrate and kept moist could not reliably provide complete infiltration of monomers into the demineralized substrate as confirmed by the degraded interfacial layer after chemical challenge (Figure 6). The porous demineralized layer provided a pathway for the basic dye [10, 14, 23] and hence for the lactic acid to penetrate [12]. The very thin and degraded interfacial layer after chemical challenge of Clearfil Protect Bond (Figure 7), the self-etch adhesive, implied that bonding monomers penetrated through the smear layer to form a hybridized smear layer was quite low; thus, smears remained and provided a leakage pathway as occurred in non-adhesive amalgam specimens. Fluoride-releasing properties in this self-etch adhesive has no influence to protect the tooth from secondary caries.

The significantly higher distance of leakage at the tooth-resin interface than that of dye penetration into the adjacent tooth surface demonstrated that lactic acid could diffuse into leakage pathways, i.e., smear layer, gap, or demineralized tooth substrate (tooth-cured adhesive interphase after phosphoric acid etching), faster than to the normal adjacent tooth surface (Table 2, Figure 3). The impermeable hybridized dentin with a leakage-free interface prepared by Super-Bond D-Liner II Plus or AQ-Bond could resist demineralization with lactic acid better than that to the adjacent normal root surface (Figure 2). This suggests that caries associated with restorations which initially occurs at the leakage pathway can be inhibited by the stable hybrid layer with the leakage-free tooth-resin interface.

## 5. Conclusions

Complete infiltration of resin into either air-dried, non-collapsed, 10–3 demineralized tooth substrate, or smear removal intact tooth surface provides the hybridized layer and tags that could resist the chemical challenge and lactic acid penetration better than that of the normal enamel and dentin. Tooth-colored restorations combined with this leakage-free hybrid layer are more reliable to protect the tooth from secondary caries than the non-adhesive amalgam and leaked resin-composite fillings. Without secondary caries, restored teeth may be maintained for life-long function and esthetics.

## Figures and Tables

**Table 1:** The composition and bonding technique of adhesive materials.

Adhesive system	Composition	Bonding technique
Self-etch	Monomers: MMA, 4-META, UDMA, 2-hydroxyethyl methacrylate, acetone, water	Scrubbed with monomer-soaked sponge on tooth surface for 20 s, air-dried 5 s, once more scrubbed, air-dried 10 s, light-cured 10 s
AQ-Bond (Touch & Bond)	Sponge: polyurethane foam, *p*-TSNa	Bulk filled with resin-composite, light-cured 60 s

Self-etch	Primer: 10-MDP, 12 MDPB, HEMA, hydrophilic dimethacrylates, water	Applied primer for 20 s, air-dried 5 s, applied monomers, air-dried 5 s, light-cured 10 s
Clearfil Protect Bond (Clearfil SE Protect)	Monomers: MDP, Bis-GMA, HEMA, hydrophobic dimethacrylate, N, N-diethanol-p-toluidine, silanated colloidal silica, surface-treated sodium fluoride	Bulk filled with resin-composite, light-cured 60 s

Moist bonding	Etchant: 32% H_3_PO_4_, water	Applied etchant for 15 s, rinsed off 15 s, air-dried 2 s, kept moist
All-Bond 2	Primer: 2% NTG-GMA, 16% BPDM, acetone	Mixed primer 1 : 1 drop for 3 s, 5 coatings on tooth surface, air-dried 5 s, light-cured 20 s
	Bonding: bis-GMA, UDMA, HEMA	Applied bonding agent and bulk filled with resin-composite, light-cured 60 s

Dry bonding	Etchant: 10% citric acid, 3% FeCl_2_ (10–3), water	Applied etchant for 10 s, rinsed off 10 s, air-dried 10 s
Super-Bond D-Liner II PLUS (Amalgam Bond Plus)	Monomers: 2, 2-bis[4-(methacryloxy polyethoxy) phenyl]propane, HEMA, 4-META, MMA, TBB	Mixed 2 drops of monomer:1 drop TBB, applied on tooth surface using brush dip technique, autocured
	Powder: PMMA	Bulk filled with amalgam

Abbreviations: MMA = methyl methacrylate; 4-META = 4-methacryloyloxyethyl trimellitate anhydride; UDMA = urethane-dimethacrylate; *p*-TSNa = amine-*p*-toluenesulfonic acid sodium salt; 10-MDP = 10-methacryloyloxydecyl dihydrogen phosphate; 12 MDPB = 12 methacryloyloxydecyl pyridinium bromide; HEMA = 2-hydroxyethyl methacrylate; bis-GMA = bisphenol A-glycidyl methacrylate; NTG-GMA = *N*-tolylglycine-glycidyl methacrylate; BPDM = biphenyl dimethacrylate; TBB = tri-*n*-butyl borane; PMMA = poly(methyl methacrylate).

**Table 2:** The distance of dye penetration into the adjacent tooth surface and the leakage at the tooth-resin interface for all groups (mean ± SD in mm).

Bonding adhesives (*n*)	Adjacent tooth surface		Leakage at tooth-resin interface	
	Enamel	Cementum/dentin	Enamel	Cementum/dentin
Sup in artificial saliva (10)	0	0	0	0
Sup in lactic acid buffer (10)	0	0.185 ± 0.045^−^	0	0
AQ in lactic acid buffer (10)	0	0.153 ± 0.030^−^	0	0
All in lactic acid buffer (10)	0	0.185 ± 0.032^−,+^	0.181 ± 0.121^a^	0.713 ± 0.929^b,+^
Cle in lactic acid buffer (10)	0	0.189 ± 0.036^−,+^	0.061 ± 0.117^a^	0.840 ± 0.894^b,+^
Non in lactic acid buffer (8)	0	0.156 ± 0.044^−,+^	0.342 ± 0.339^a^	0.558 ± 0.222^b,+^

0 = no dye penetration or no leakage. ^−^ No significant difference between groups (*p* > 0.05). ^+^Significant difference between groups in the same row (*p* < 0.05). ^a,b^Significant difference between groups with different superscripts in the same row and colume (*p* < 0.05).

## Data Availability

Raw data can be requested from the corresponding author.
